# Plasmon-Enhanced Photocatalytic CO_2_ Reduction for Higher-Order Hydrocarbon Generation Using Plasmonic Nano-Finger Arrays

**DOI:** 10.3390/nano13111753

**Published:** 2023-05-27

**Authors:** Tse-Hsien Ou, Pan Hu, Zerui Liu, Yunxiang Wang, Sushmit Hossain, Deming Meng, Yudi Shi, Sonia Zhang, Boxin Zhang, Boxiang Song, Fanxin Liu, Stephen B. Cronin, Wei Wu

**Affiliations:** 1 Ming Hsieh Department of Electrical and Computer Engineering, University of Southern California, Los Angeles, CA 90089, USA; 2 Mork Family Department of Chemical Engineering and Material Science, University of Southern California, Los Angeles, CA 90089, USA; 3 Wuhan National Laboratory for Optoelectronics, Huazhong University of Science and Technology, Wuhan 430074, China; 4 Department of Applied Physics, Zhejiang University of Technology, Hangzhou 310023, China; 5 Department of Chemistry, University of Southern California, Los Angeles, CA 90089, USA

**Keywords:** CO_2_ reduction, photocatalysis, nano-fingers, plasmon, higher-order hydrocarbon

## Abstract

The carbon dioxide reduction reaction (CO2RR) is a promising method to both reduce greenhouse gas carbon dioxide (CO_2_) concentrations and provide an alternative to fossil fuel by converting water and CO_2_ into high-energy-density chemicals. Nevertheless, the CO2RR suffers from high chemical reaction barriers and low selectivity. Here we demonstrate that 4 nm gap plasmonic nano-finger arrays provide a reliable and repeatable plasmon-resonant photocatalyst for multiple-electrons reactions: the CO2RR to generate higher-order hydrocarbons. Electromagnetics simulation shows that hot spots with 10,000 light intensity enhancement can be achieved using nano-gap fingers under a resonant wavelength of 638 nm. From cryogenic ^1^H-NMR spectra, formic acid and acetic acid productions are observed with a nano-fingers array sample. After 1 h laser irradiation, we only observe the generation of formic acid in the liquid solution. While increasing the laser irradiation period, we observe both formic and acetic acid in the liquid solution. We also observe that laser irradiation at different wavelengths significantly affected the generation of formic acid and acetic acid. The ratio, 2.29, of the product concentration generated at the resonant wavelength 638 nm and the non-resonant wavelength 405 nm is close to the ratio, 4.93, of the generated hot electrons inside the TiO_2_ layer at different wavelengths from the electromagnetics simulation. This shows that product generation is related to the strength of localized electric fields.

## 1. Introduction

CO_2_ is one of the greenhouse gases in the atmosphere that causes global warming and rising sea levels. The global CO_2_ emissions from the burning of fossil fuels for energy, however, increased significantly in the past and has reached over 34 billion tons each year [1,2]. The CO2RR is a promising method to both reduce the CO_2_ concentration in the atmosphere and be an alternative fuel source as it not only can mitigate the CO_2_ concentration but also can convert water and CO_2_ into high-energy-density chemicals such as carbon monoxide (CO), methanol, and ethylene [3,4,5,6,7]. Converting CO_2_ with the water molecule (H_2_O) to various hydrocarbons, however, is a complicated reaction system, as it involves multiple electrons, requires up to 13 electrons and up to 8 protons to generate various hydrocarbons, and needs to compete with the hydrogen (H_2_) evolution reaction, which has a low reduction potential, for electrons and protons [8,9,10]. The standard electrode potentials of different CO2RRs with reference to the normal hydrogen electrode (NHE) for different produces in aqueous solutions are summarized in Table 1 [11,12]. A slush reaction rate of the CO2RR due to high chemical reaction barriers, the competition with hydrogen (H_2_) evolution for protons and electrons, and low selectivity to generate higher-order hydrocarbons greatly hinder its application [8,13,14,15,16]. Many research groups have put much effort into the electrochemical, photoelectrochemical, or photocatalytic CO2RR, including using various plasma discharge approaches [17,18,19], various plasmonic nanoparticles (NPs) [8,20], and plasmon-enhanced noble NP decorated electrodes [8,21,22,23] to generate higher-order hydrocarbons. However, these methods either require extra external energy (e.g., voltages or currents), involve sophisticated material preparations to synthesize plasmonic nanoparticles, or involve uncontrollable shapes of plasmonic nanoparticles. Yang et al. used transient pulsed plasma to generate higher hydrocarbons from the CO2RR using a 10 mL carbonated solution with pulses with up to 30 kV peak voltages, pulse rise time of 5–10 ns, and repetition rates up to 2 kHz for 30 min of plasma discharge [19]. Creel et al. deposited a layer of 200 nm Ag on a clean glass slide as a cathode to study electrochemical CO2RR using plasmonics. From their Scanning electron microscopy (SEM) images and atomic force microscopy (AFM) topographic images, they observed nodule-like features of 10−100 nm on the electrode surface. These nodule-like features generated plasmonics under a 365 nm wavelength to help generate carbon monoxide (CO), formate, and methanol while suppressing the generation of H_2_ [8]. Corson et al. electrochemically formed Cu nanocorals nanostructure on an Ag cathode to study electrochemical CO2RR using plasmonics. They showed that the sharp features given from the nanocoral morphology can serve to enhance the plasmonics and therefore enhance the generation of higher hydrocarbons from CO2RR [23]. For the photocatalytic CO2RR using plasmonics, Hou et al. deposited a 5 nm thick gold film on a TiO_2_ film to study the CO2RR. The 5 nm thick gold film was not thick enough to form a continuous film. Instead, this film formed different islands that could be seen as nanoparticles for generating plasmon [24].

Among these methods, the photocatalysis of the CO2RR has gained great interest due to its ability to convert light energy to reduce CO_2_ to generate higher-order hydrocarbons without applying any external electricity [24,25,26,27]. To make photocatalysts, titanium dioxide (TiO_2_) is one of the widely used materials due to its high chemical stability, availability, and environmental friendliness. However, TiO_2_ suffers from its low quantum efficiency due to its fast recombination rate of generated electrons and holes and its wide band gap (3.2 eV), which greatly limits its photocatalytic efficiency under visible light wavelengths [15,25,26]. According to our previous studies, our 4 nm gap plasmonic nano-finger arrays provide a hot spot intensity with a light energy enhancement 10,000 times larger than a 2 nm TiO_2_ film sample at the resonant wavelength near 600 nm [25,27]. Within our plasmonic nano-fingers, a layer of 4 nm TiO_2_ in total is located at the hot spot locations, and this 4 nm TiO_2_ layer greatly reduces the probability of electron-hole pairs being recombined before reaching to the catalyst surface to perform reduction and oxidation reactions. In addition, an ultra-high hot spot intensity generated near 600 nm wavelength allows TiO_2_ to have energetic electrons and holes to perform reduction and oxidation reactions under the visible light wavelengths that greatly extends TiO_2_′s applications in photocatalysis [8,21,23]. Furthermore, some recent studies have reported that the plasmonic effect helps suppress H_2_ evolution and yield higher-order hydrocarbons, although the detailed mechanisms remain unclear [8,21,23]. However, most of the current progress on the photocatalysis of the CO2RR still involves many complicated chemical syntheses and the uncontrollable shapes of plasmonic nanoparticles [24,28,29,30]. Plasmonic nanoparticles are usually randomly synthesized, decorated, or formed on an electrode or a substrate. The location and the shape of the plasmonic nanoparticles are not controllable, so the hot spot intensity and the resonant wavelength of the plasmonic nanoparticles are not controllable as well. Due to the uncertainty on the hot spot intensity and location, the performance of the photocatalysis of the CO2RR might not be repeatable.

In this article, we proposed using a nano-finger array as a platform of photocatalysis to perform the CO2RR. A nano-finger array was fabricated via nanoimprint lithography (NIL). This nano-finger array platform can provide several advantages: (1) simplified fabrication processes for making plasmonic nanoparticles; (2) a controllable, consistent geometry of plasmonic nanostructures; and (3) allowing quantitative analysis on the photocatalysis of the CO2RR due to well-defined hot spot’s location. A controllable and consistent geometry of plasmonic nanostructures allow us to have consistent and periodic ultra-high hot spot intensities under a target resonant wavelength across a nano-finger array sample. An air-tight reaction cell was used to form a CO_2_-saturated deionized (DI) water solution to study the CO2RR and to collect liquid samples after the CO2RR. A nano-finger array sample under a resonant wavelength laser irradiation with different irradiation periods was studied. The sample under different laser irradiation wavelengths was used to study the relationship between the product generations and the hot spot intensity. ^1^H-NMR measurements were performed to identify chemicals and their concentrations in liquid solutions after the CO2RR. Electromagnetics simulation was used to model the hot spot intensity generated using nano-fingers and to study the relationship between the product concentrations and the hot spot intensity. With a high hot spot intensity, the consistent geometry of plasmonic nanostructures, and the ability to quantitatively analyze the photocatalysis of the CO2RR, our 4 nm gap plasmonic nano-finger array can be a reliable and repeatable platform for not only performing photocatalysis on the CO2RR that satisfies the need to perform the CO2RR for mitigating CO_2_ concentration in the atmosphere and be an alternative fuel source but also a good way to understand how optical excitation contributes to the CO2RR.

## 2. Materials and Methods

### 2.1. Fabrication of the Device

**Figure 1:**
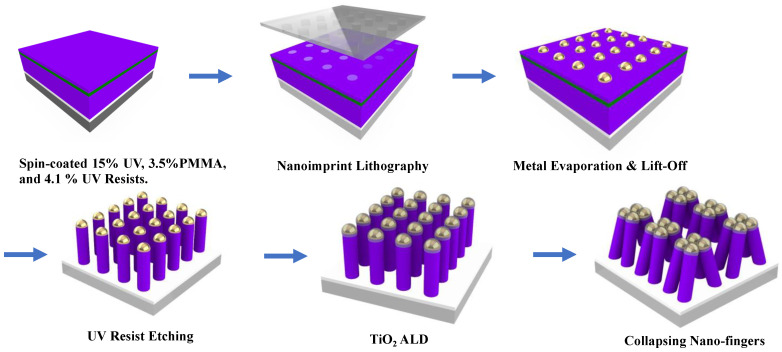
Schematic fabrication processes for a nano-finger array.

The schematic fabrication processes for a nano-finger array are shown in Figure 1. Hydrophilic surface treatment was first applied on a 3-inch silicon dioxide substrate to increase the adhesion between the substrate and the following first UV resist layer. After the surface treatment, a layer of 600 nm 15% UV nanoimprint resist (EZImprinting Inc., Los Angeles, CA, USA) was spin-coated on the substrate at 1500 rpm for 30 s. Before UV curing, nitrogen gas was purged into the curing chamber for 20 min to minimize any degraded UV film caused by oxygen (O_2_) in the air. A UV lamp with a power of 4 mW/cm^2^ and a wavelength of 365 nm was therefore used to cure the UV resist for 2 min under the nitrogen ambient. A layer of 100 nm 3.5% PMMA (MilliporeSigma, Munich, Germany) dissolved in anisole (MilliporeSigma, Munich, Germany) as a lift-off layer was spin-coated at 4000 rpm for 60 s on the 15% UV resist, followed by baking at 180 °C for 10 min. A layer of 100 nm 4.1% UV nanoimprint resist (EZImprinting Inc.) was spin-coated at 2500 rpm for 10 s on the lift-off layer. NIL was performed to form a two-dimensional hole array on the 4.1% UV resist layer. The residual layer was then etched using RIE (PlasmaPro RIE 80, Oxford Instruments, Abingdon, Oxfordshire, UK) to expose the bottom 15% UV resist layer. A layer of 2 nm Ti (99.995%, Kurt J. Lesker Company, Jefferson Hills, PA, USA) as an adhesion layer and a layer of 50 nm Au (99.999%, Kurt J. Lesker Company, USA) was evaporated via an e-beam evaporator (PRO Line PVD 75, Kurt J. Lesker Company, USA) on the sample. In the lift-off process, an acetone bath at room temperature for 10 min was used to dissolve the PMMA layer (i.e., lift-off layer) to remove undesired Au films. After the lift-off process, an Au nanoparticle array on the 15% UV resist layer was left. The sample was then etched using RIE to achieve a high-aspect-ratio nano-fingers array. For each nano-finger, there is an Au nanoparticle on top of a 15% UV resist pillar. A 2 nm amorphous TiO_2_ film was deposited on the nano-fingers with a growing temperature of 100 °C using a plasma-enhanced ALD (Ultratech Simply ALD, Veeco) to encapsulate the Au nanoparticles on top of the 15% UV resist pillars. Finally, the nano-fingers were soaked in ethyl alcohol 200 proof solution (Decon Labs, King of Prussia, PA, USA) and then air-dried at room temperature. The capillary force from ethyl alcohol evaporation leads to adjacent nano-fingers collapse together.

### 2.2. Characterization

Scanning electron microscopy (SEM) images of the nano-fingers before and after collapsing were taken using Nova NanoSEM 450 (Field Electron and Ion Company, Hillsboro, OR, USA) under an accelerating voltage 10 kV with a spot size 3. To demonstrate there is a layer of 4 nm TiO_2_ between collapsing nano-fingers, transmission electron microscopy (TEM) and energy-dispersive X-ray spectroscopy (EDS), JEOL JEM-2100F (JEOL Ltd., Tokyo, Japan) were used for cross-sectional analysis. For TEM cross-sectional view sample preparation, dual beam focus ion beam (FIB), Seiko 4050MS (Seiko Instruments Inc., Tokyo, Japan) was used to cut the nano-fingers to expose the cross-section of nano-fingers.

Cryogenic ^1^H-NMR measurements were performed using Bruker Avance III HD 800 MHz NMR equipped with TCI cryoprobe. The 5 mm with 7-inch-long NMR sample tubes (NE-UP5-7, NewEra) were used in the measurement. Each NMR tube includes 10 µL 21 mM DMSO-d6 (D, 99.9%, Cambridge Isotope Laboratories, Inc., Tewksbury, MA, USA), 10 µL deuterium oxide (D_2_O, D, 99.9%, Cambridge Isotope Laboratories, Inc., Tewksbury, MA, USA), and 190 µL test solution to form a one mM DMSO-d6 test solution. The NMR data were collected using a water suppression pulse program (excitation sculpting with gradients) at 25 °C. A spectral width of 12,829 Hz (16 ppm) was used along with relaxation delay of 1 s and 128 scans for each sample. The MestReNova software was used to analyze the NMR spectra. The reference peak was chosen at chemical shift 2.5 ppm to represent the reference chemical DMSO. The automatic phase correction and automatic baseline correction functions were used to correct the phase and baseline in each spectrum.

Fourier-transform infrared (FTIR) measurements were performed via an Agilent Cary 630 FTIR Spectrometer equipped with a diamond ATR. Before each measurement, the crystal on the diamond ATR was cleaned with acetone, and the spectrometer was calibrated. After the calibration, two droplets of a liquid sample were dropped on the crystal. Rotating a sample press knob to make a sample press tip closely contact with the crystal. Each spectrum was measured from wavenumber 650 cm^−1^ to 4000 cm^−1^ with an interval 2 cm^−1^.

The pH value of DI water or CO_2_-saturated DI water was measured using Apera Instruments AI311 Premium Series PH60 Waterproof pH Pocket Tester with ±0.01 pH accuracy. Before measurements, the pH tester was calibrated using calibration buffer solutions with 3 different pH values (i.e., 4, 7, and 10). Before measuring the pH value of DI water or CO_2_-saturated DI water, the pH tester was rinsed with DI water and air dried with N_2_ gas. During the measurement, the sensor of the pH tester was immersed in 10 mL of DI water or CO_2_-saturated DI water. The pH value was recorded when the pH value displayed on the pH tester was stable.

### 2.3. Electromagnetics Simulation

A commercial electromagnetics simulation software, Lumerical Finite-difference time-domain (FDTD), was used to model the electric field distribution near the nano-fingers. In the modeling, the nano-finger consisted of a UV resist cylinder capped with a 50 nm thick Au nanoparticle and coated with a 2 nm TiO_2_ film. The shape of the hemisphere was used to represent the Au nanoparticle in the modeling to approximate the actual shape of Au nanoparticle observed in the cross-sectional SEM images as shown in Figure S1a in our previous work [27]. The light intensity enhancement with different cross-sectional geometries of nanoparticles does not change significantly as shown in Figure S1b. The diameter and height of the nano-finger were 60 nm and 350 nm, respectively. Two nano-fingers were tilted toward each other so that they were in close contact with each other. The refractive indices of TiO_2_ and UV resist were measured using VAS Ellipsometer (J.A. Woollam, Lincoln, NE, USA). The measured refractive indices of TiO_2_ and UV resist were imported into modeling for accurate modeling results. The refractive index of Au was obtained from Johnson and Christy [31]. The background refractive index was set as 1.33 to approximate the nano-fingers immersed in a water solution environment. The nano-finger was placed on top of an infinite silicon dioxide substrate and excited with a plane wave with a range of wavelengths from 550 nm to 650 nm at normal incidence.

## 3. Results and Discussion

**Figure 2:**
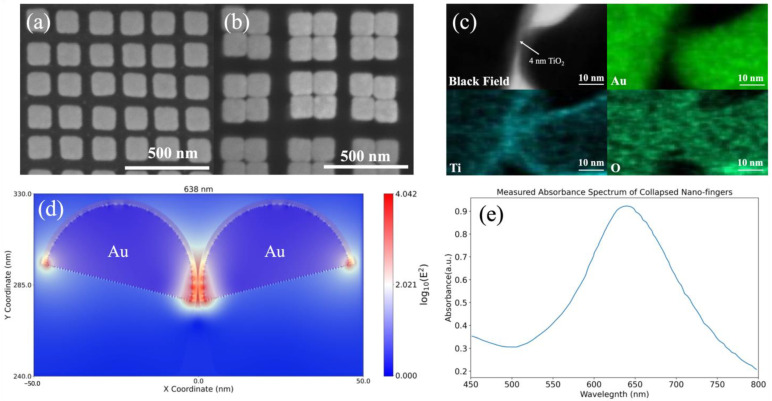
SEM image of the nano-finger array (**a**) before collapsing and (**b**) after collapsing; (**c**) TEM image of the nano-gap fingers and its EDS mapping of Au, Ti, and O; (**d**) Light intensity distribution of the nano-gap fingers from the electromagnetics simulation; (**e**) Measured absorbance spectrum of a collapsed nano-finger array from the electromagnetics simulation.

In this study, we demonstrate that our 4 nm gap plasmonic nano-finger array made via the nanoimprint technique with an ultra-high hot spot intensity can be a reliable, repeatable plasmonic photocatalyst for the CO2RR to generate higher-order hydrocarbons that contain high-energy density at visible wavelength 638 nm. The SEM images of nano-fingers before and after collapsing are shown in Figure 2a,b, respectively. The diameter and height of each nano-finger are 60 nm and 350 nm, respectively, and the pitch is 200 nm. The capillary force due to the evaporation of ethyl alcohol draws adjacent nano-fingers to collapse together. Once the nano-fingers come together, they will not separate due to van der Waals force [27,32]. With the NIL technique, the fabrication of 4 nm gap plasmonic nano-finger array is reliable and repeatable. A layer of 2 nm TiO_2_ is conformally deposited via ALD on each nano-finger. After the nano-fingers collapsed, there is a 4 nm TiO_2_ layer in total between each nano-finger. The reason of choosing a layer of 4 nm TiO_2_ layer is that from our previous works, a 4 nm TiO_2_ gap can provide the strongest field enhancement [25,27]. The TEM image and the EDS mapping of Au, Ti, and O in the TEM image in Figure 2c confirm that nano-fingers have a 4 nm TiO_2_ gap between Au NPs: in black field image and Au mapping, there is a gap between each NPs while in Ti and O mappings two NPs are connected. From our Lumerical FDTD simulation shown in Figure 2d, the hot spots of nano-fingers with light intensity are 10,000-fold larger than the incident light intensity are generated under 638 nm incident wavelength. Although the Au NPs seems to be squared-shaped nanostructures in the SEM images, we find that the shape of Au NPs is more like the hemisphere in our previous works [27,33] as mentioned in the material and method section and as shown in Figure S1a. In addition, the light intensity enhancement does not change significantly when using squared-shaped nanostructures in the modeling as shown in Figure S1b. Therefore, we used the shape of hemispheres to represent Au NPs in our nano-fingers to better represent what we have observed from the cross-sectional SEM image. The collapsed nano-fingers form a cavity with an only 4 nm gap distance, which produces gap plasmon in the middle of nano-fingers. The gap plasmon resonated between two Au NPs, therefore producing a 10,000-fold light intensity enhancement compared to the incident light intensity. Note that, under 638 nm, the extinct coefficient of Au is low [31] and the refractive index of TiO_2_ (2.58) [34] is larger than the refractive index of water (1.33) [35]; light can be trapped inside the TiO_2_ layer, so the light intensity can be expected inside the TiO_2_ layer. Due to high light intensity enhancement from the hot spots of the nano-fingers, strong light intensities inside the TiO_2_ layer can be observed from the simulation results shown in Figure 2d. Figure 2e shows the measured absorbance spectrum of collapsed nano-fingers with a resonant peak near 650 nm, indicating that using a 638 nm laser source can generate hot spots in nano-fingers.

**Figure 3:**
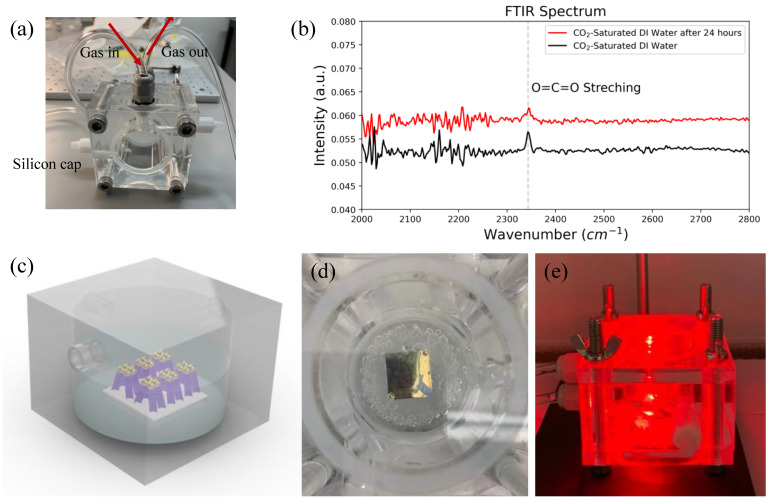
(**a**) Air-tight PMMA reaction cell used for the CO2RR in this study; (**b**) FTIR spectra of fresh CO_2_-saturated DI water and CO_2_-saturated DI water after 24 h; (**c**) Schematic diagram of the air-tight reaction cell with a nano-finger array soaked in 25 mL CO_2_-saturated DI water; (**d**) Photograph of a nano-finger array immersed in 25 mL CO_2_-saturated DI water with many CO_2_ bubbles; (**e**) Photograph of a 638 nm laser focused on a nano-finger array placed inside the reaction cell with 25 mL CO_2_-saturated DI water.

To study the CO2RR, an air-tight Poly(methyl methacrylate) (PMMA) reaction cell, as shown in Figure 3a, is used to purge CO_2_ gas into DI water to form CO_2_-saturated DI water and to collect liquid products for the following chemicals characterization. Some reasons of choosing a PMMA-based reaction cell are that the PMMA reaction cell is inexpensive, has low absorptions in visible light spectra, and is robust compared with glass-based reaction cells. A total of 25 mL CO_2_-saturated DI water solutions were prepared by purging 99.999% CO_2_ gas into DI water for 30 min at 1 atm at 25 °C before each experiment. To minimize any CO_2_ in the atmosphere dissolved into DI water to affect the experiments, fresh DI water with a measured 6.41 pH value was used to prepare CO_2_-saturated DI water. After purging CO_2_ into DI water for 30 min, a measured pH value of DI water became 3.92 pH value indicating that CO_2_ was dissolved in DI water [36,37]. The initial CO_2_ dissolved into DI water was estimated to be around 1.4 g/L [38] (p. 5.5), which corresponded to 31.8 mM. The Fourier-transform infrared (FTIR) spectrum was used to study whether CO_2_ gas dissolved in DI water. Since the dissolvability of CO_2_ in DI water is low under 1 atm and room temperature [38], to better analyze whether there are CO_2_ peaks in the FTIR spectrum, we subtract the spectrum of DI water from the spectrum of CO_2_-saturated DI water, as shown in Figure 3b. In Figure 3b, a peak at wavenumber 2349 cm^−1^ appeared in a black line corresponds to a CO_2_ stretching peak [39], which confirms that CO_2_ has dissolved in DI water, and the solution can be used for studying the CO2RR. The red line is the CO_2_-saturated DI water solution stored inside the reaction cell for 24 h, a CO_2_ stretching peak can still be observed from the FTIR spectrum indicating the amount of CO_2_ inside the solution is rich enough for at least 24 h of laser irradiation experiments. Figure 3c is a schematic figure illustrating that the nano-finger array soaks in a 25 mL CO_2_-saturated DI water solution in an air-tight reaction cell. Figure 3d shows that after forming a CO_2_-saturated DI water solution, many bubbles formed inside the solution. Lasers with 638 nm and 405 nm wavelengths with power 300 mW (MXTLASER Corporation, Zhongshan, China) were used in this study, and a laser with 638 nm wavelength was focused on the nano-finger array with a laser spot size diameter around 10 mm, as shown in Figure 3e.

**Figure 4:**
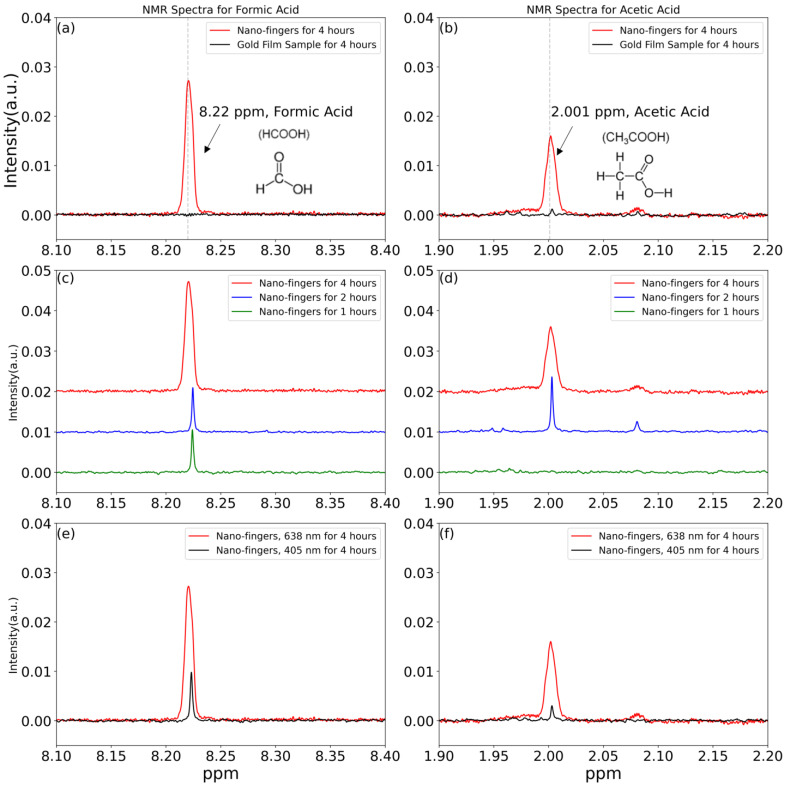
1H-NMR spectra of liquid samples (**a**,**b**) with a nano-finger array and a gold film sample; (**c**,**d**) with a nano-finger array after different irradiation times; (**e**,**f**) with a nano-finger array under different incident wavelengths.

As mentioned in the introduction, the CO2RR is a complicated reaction system as it involves multiple electrons to generate higher-order hydrocarbons. Our 4 nm gap plasmonic nano-finger array with an ultra-strong hot spot intensity can generate many hot electrons in Au NPs that can generate higher-order hydrocarbons from the CO2RR. A sample size of 1 cm by 1 cm nano-finger array soaked in a 25 mL CO_2_-saturated DI water solution with an optical excitation was used to study the CO2RR. To identify the products that were generated after the CO2RR and to know the concentration of each product, cryogenic ^1^H-NMR measurements with a known concentration of 1 mM dimethyl sulfoxide-d6 (DMSO-d6) were performed. To better compare the intensity of each product peak in different experiments, Figure 4a–f are normalized ^1^H-NMR spectra with respect to the intensity of 1 mM DMSO-d6. The CO2RR with a sample size of 1 cm by 1 cm nano-finger array and the CO2RR with a sample size of 1 cm by 1 cm of a 50 nm gold film substrate under 638 nm laser irradiation for 4 h were studied, as shown in Figure 4a,b. From the literature, formic acid (CH_2_O_2_) has a single peak with chemical shift around 8.22~8.33 ppm, and acetic acid (CH_3_COOH) has a single peak with chemical shift around 1.79~2.08 ppm in ^1^H-NMR under D_2_O solvent conditions [19,40,41,42]. In Figure 4a, there is a clear peak at the chemical shift of 8.22 being ppm shown in a red line, indicting that there is formic acid formed in CO_2_-saturated DI water after using a nano-finger array sample for 4 h of 638 nm laser irradiation. However, there is no peak (black line) observed at the chemical shift of 8.22 ppm, indicating no formation of formic acid after 4 h of laser irradiation with gold film sample. In Figure 4b, we also observe there is a clear peak (red line) at the chemical shift of 2.0 ppm, indicting that the formation of the acetic acid in CO_2_-saturated DI water with a nano-finger array sample after the laser irradiation. Again, there is no peak (black line) at the same chemical shift position, indicating no formation of acetic acid after the laser irradiation with gold film sample. Our latest research on using nano-fingers to photo-decompose methyl orange (MO) also shows that a 2 nm TiO_2_ film could not perform observable photocatalysis even after 9 h of 532 nm laser irradiation [25]. This indicates that the hot spots generated using nano-fingers facilitate the photocatalysis and can generate higher-order hydrocarbons from the CO2RR in our case. The CO2RR with different irradiation times was also studied, as shown in Figure 4c,d. Comparing Figure 4c,d, only the peak of formic acid was observed with 1 h of laser irradiation (green line). However, after 2 h (blue line) and 4 h (red line) of laser irradiation, both peaks of formic acid and acetic acid can be observed, and the intensity of peaks increases as the laser irradiation time increases. We also studied the CO2RR with different incident wavelengths as shown in Figure 4e,f. Comparing Figure 4e,f, a resonant wavelength (638 nm, red line) and a non-resonant wavelength (405 nm, black line) both have two clear peaks that indicate the formation of formic acid and acetic acid in CO_2_-saturated DI water with a nano-finger array sample after the 4 h of laser irradiation. However, we noticed that when using the resonant wavelength, the intensities of peaks for formic acid and acetic acid were higher than those for formic acid and acetic acid when using the non-resonant wavelength. Since gold has a larger imaginary part of the epsilon at 405 nm than at 638 nm, as shown in Figure S2 [31], most of the incident light energy is absorbed by gold at 405 nm, which results in weaker hot spots, as shown in Figure S3. Note that, comparing Figure S3 with Figure 2d, the light intensities inside the TiO_2_ layer are also weaker. Therefore, this indicates that the intensity of hot spots affects the generation rate of formic acid and acetic acid.

**Figure 5:**
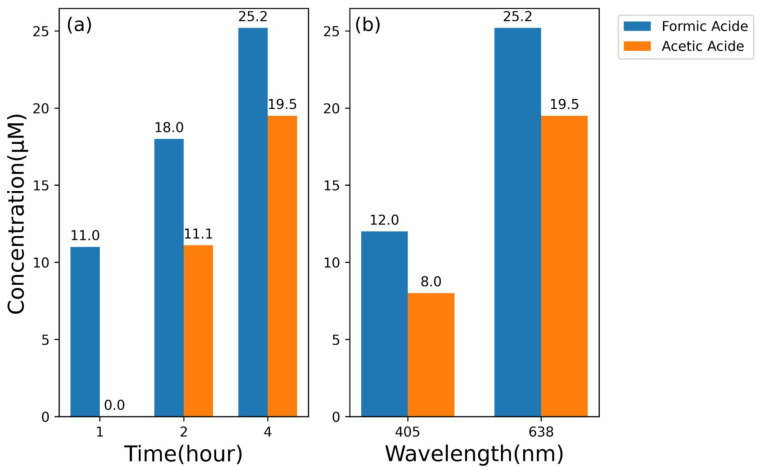

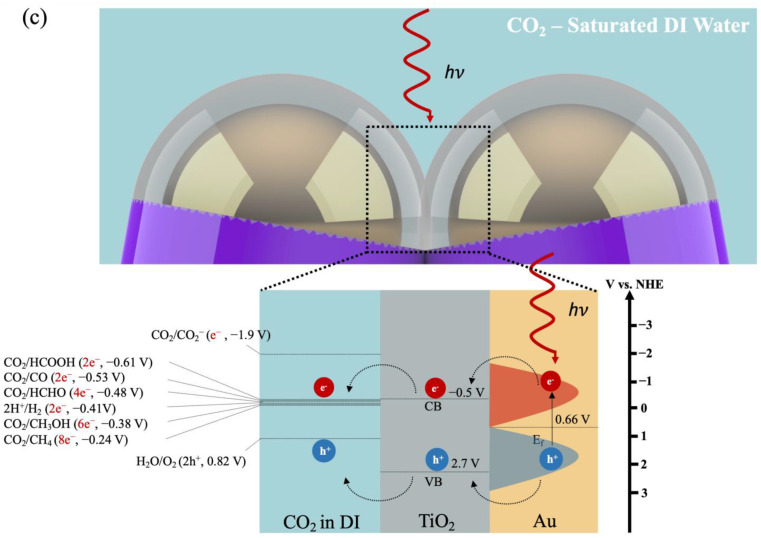
(**a**) Concentrations of formic acid and acetic acid observed under different irradiation times; (**b**) Concentrations of formic acid and acetic acid under different incident wavelengths; (**c**) Schematic diagram illustrating the hot carrier energy transfer mechanism [10,11,12,46,47,48,52].

The normalized ^1^H-NMR spectra are also convenient for us to calculate each product concentration with respect to a known concentration of 1 mM DMSO-d6; therefore, we further integrate the peak area of each product peak to find their corresponding concentration. In Figure 4c,d, 11 µM of formic acid but no acetic acid was generated after 1 h of 638 nm laser irradiation, 18 µM of formic acid and 1.1 µM of acetic acid were generated after 2 h of 638 nm laser irradiation, and 25.2 µM of formic acid and 19.5 µM of acetic acid were generated after 4 h of 638 nm laser irradiation. We plotted this dependence of product concentrations with different irradiation times in Figure 5a for easy observation. From our results, in our case with 638 nm laser irradiation, we observe that the product concentrations increase roughly linearly as the irradiation time increases, and acetic acid generation starts after 1 h of laser irradiation. However, the wavelength of the light source used to excite plasmons and the geometrical shape of plasmonic nanostructures greatly affect the orientation of chemicals absorbed on the plasmonic NPs, which therefore affect the chemical reaction and the generation of chemicals [28]. This tendency might change if we use different wavelengths or different geometrical plasmonic nanostructures. Some studies have shown that acetic acid can be generated from formic acid or oxalate although a detailed pathway is not currently understood yet. To form oxalate, one of the potential reaction pathways is CO_2_ + e^−^ → CO_2_^−^ and then followed by CO_2_^−^ + CO_2_ → (CO_2_^−^)_2_ [19,36,43,44]. To better understand the potential needed for chemical reactions, the energy from the absolute scale [unit given in electron Voltage] is converted to the reduction potential versus normal hydrogen electrode (NHE) [unit given in Voltage]: the energy in absolute scale is 4.44 eV more positive to the reduction energy with reference to NHE [in electron Voltage] [45]. From Table 1, since the reduction potential (−1.9 V vs. NHE) of CO_2_ + e^−^ → CO_2_^−^ is 1.4 V more negative than the conduction band of the TiO_2_ (−0.5 V vs. NHE) [46,47,48] shown in Figure 5c, an additional 1.4 V is required for electrons inside TiO_2_ layer to reduce CO_2_ to form CO_2_^−^ and therefore form oxalate [13,16,26,49]. Accordingly, we believe that the acetic acid generated in our experiments is not from oxalate. Instead, the reduction potential to form formic acid (−0.61 V vs. NHE) is more positive than the conduction band of the TiO_2_; therefore, we believe that the acetic acid in our case is mainly generated from formic acid with the following reactions: HCOO^−^ + 5H^+^ + 4e^−^ → CH_3_OH + H_2_O and HCOO^−^ + CH_3_OH + H^+^ → CH_3_COOH + H_2_O (2HCOO^−^ + 6H^+^ + 4e^−^ → CH_3_COOH + H_2_O) [19]. Since two formic acids can convert to one acetic acid, we can calculate the average yield of formic acid, which is about 12.38 µM per hour, from our ^1^H-NMR results. To analyze the effect of different incident wavelengths on product generations, we integrate the peak area of each product peak in Figure 4e,f to find their corresponding concentration. There were 25.2 µM of formic acid and 19.5 µM of acetic acid formed after 4 h of 638 nm laser irradiation, which corresponds to the yield of formic acid of about 16.05 µM per hour. While for 4 h of 405 nm laser irradiation, there were 12 µM of formic acid and 8 µM of acetic acid formed, which corresponds to the yield of formic acid of about 7 µM per hour. In Figure 5b, we plotted this dependence of product concentrations with different irradiation wavelength for easy observation. We observed that under the same laser irradiation time, 4 h, both the formic acid and acetic acid concentrations under a resonant wavelength (638 nm) are higher than those under a non-resonant wavelength (405 nm). As mentioned before, we believed that the hot spots intensity of nano-fingers generated at different wavelengths affects the product generations. The yield of formic acid at 638 nm laser irradiation is 2.29 times larger than that of formic acid at 405 nm. This indicates that the strong hot spot intensity helps the CO2RR product generation even though the photon energy of 405 nm (3.06 eV) is larger than the photon energy of 638 nm (1.94 eV).

To explain why nano-fingers can enhance the CO2RR, the energy transfer mechanism of hot carriers inside Au NPs to the TiO_2_ layer upon the laser irradiation is illustrated in Figure 5c. When incident light with the resonant wavelength impinges on the Au NPs, a nonthermal distribution of hot carriers is generated inside Au NPs. In our case, under the 638 nm excitation, energetic hot electron distribution with energy up to 1.94 eV above the work function of Au (0.66 V vs. NHE) is generated. The hot electrons with potential −1.28 V vs. NHE can be injected into the TiO_2_’s conduction band (−0.5 V vs. NHE). These energetic hot electrons in TiO_2_’s conduction band can further react with CO_2_ in DI water to perform the CO2RR according to each reduction potential of the CO2RR listed in Table 1 as well as shown in Figure 5c. On the other hand, the same number of hot holes can inject into the TiO_2_’s valence band (2.7 V vs. NHE) and further react with H_2_O to generate oxygen (O_2_). With only 2 nm TiO_2_ thickness on each nano-finger, this thin TiO_2_ layer can largely reduce the probability of electron-hole pairs being recombined before reaching the TiO_2_ surface to perform reduction and oxidation reactions [25,27].

The FDTD simulation is used to study why the yield of products at 638 nm is more significant than that of products at 405 nm. From the simulation, the maximum light intensity enhancement factor of the hot spot at 638 nm is around 10,000 while the light intensity enhancement factor of the hot spot at 405 nm is only around 20 compared with the incident light intensity. The hot electrons that react with CO_2_ to perform the CO2RR are from the TiO_2_ layer, and the total amount of hot electrons is proportional to the light intensity coupled into the nano-fingers, implying that the generated hot electrons are proportional to the square of the localized electric fields [25,27]. Therefore, we can approximate the number of generated hot electrons via the light intensity inside the TiO_2_ layer from the FDTD simulation. Here, we estimate the ratio of the rate of hot electron generation at 638 nm to the rate of hot electron generation at 405 nm based on the light intensity inside TiO_2_ layer at the same incident intensity, assuming the hot electron generation efficiencies are the same for both wavelengths.

$$Ratio=\frac{\oint_{{volume\;of\;TiO}_{2}}^{}\frac{{\left|E\right|}^{2}}{{{|E}_{0}|}^{2}}@\;638\;\mathrm{nm}}{\oint_{{volume\;of\;TiO}_{2}}^{}\frac{{\left|E\right|}^{2}}{{{|E}_{0}|}^{2}}@\;405\;\mathrm{nm}}\times \frac{3.06\;\mathrm{eV}}{1.94\;\mathrm{eV}}$$

From the FDTD simulation, the light intensity inside the TiO_2_ layer at 638 nm is 3.13 times of the light intensity inside the TiO_2_ layer at 405 nm. This indicates that the number of hot electrons inside the TiO_2_ layer at 638 nm is 4.93 times of the TiO_2_ layer at 405 nm. Note that this ratio is related to the ratio of the actual product concentration generated at 638 nm to the product concentration generated at 405 nm, if all the hot electrons contribute to the product generation and only the CO2RR occurs. Recall that the ratio calculated from the product concentration at each incident wavelength is about 2.29. Compared with the ratio of the number of hot electrons inside the TiO_2_ layer, the lower ratio we got from the experimental results has the following possible reasons: only calculating the product concentration based on the liquid samples, the recombination of electron-hole pair in TiO_2_ and Au, and the competition for electrons and protons between H_2_ evolution [8,50,51]. However, the generated hot electron ratio and the actual product generation ratio confirm that the plasmonic effect dramatically enhances the CO2RR product generation by the increased number of hot electrons.

The reason why there are only liquid samples collected for analysis is that the product generation rate is not high enough compared with the gas leakage rate of the PMMA reaction cell. Therefore, we left the optimization of the reaction cell as our future work. However, by only analyzing the liquid samples after the CO2RR, we demonstrate that our 4 nm gap plasmonic nano-finger array with an ultra-strong hot spot intensity can generate many hot electrons in Au NPs for reactions involving multiple electrons such as the CO2RR for the generation of higher-order hydrocarbons. This discovery implies that if we can have a new type of device, a nano-fingers-like device that allows applying voltage on them, we can take advantage of ultra-strong hot spot intensity to generate many hot electrons for reactions and can increase the product generation rate with a lower applied voltage. Therefore, we also left the design and fabrication of nano-fingers-like devices in our future work.

## 4. Conclusions

In summary, we demonstrate that 4 nm gap plasmonic nano-finger array made by the nanoimprint technique with an ultra-high hot spot intensity can be a reliable, repeatable plasmonic photocatalyst for the CO2RR to generate higher-order hydrocarbons. The ultra-high hot spot intensity can generate many hot electrons in Au NPs for reactions involving multiple electrons such as the CO2RR. We observe product generation is proportional to the 638 nm laser irradiation time on the nano-finger arrays. Only a formic acid peak is observed from cryogenic ^1^H-NMR spectroscopy after 1 h of 638 nm laser irradiation. However, both formic and acetic acid peaks can be observed after 2 h and 4 h of 638 nm laser irradiation. We explained that acetic acid generated in this study is mainly from formic acid, and the average yield of formic acid is about 12.38 µM per hour. We also use a non-resonant wavelength (405 nm) on nano-finger arrays to study the product generation of the CO2RR. The product generation rate under 638 nm is about 2.29 times the product generation rate under 405 nm. The ratio (4.93) estimated from the FDTD simulation, and the ratio (2.29) calculated from the product generation shows the relationship with the light intensity, confirming that the plasmonic effect dramatically enhances the CO2RR product generation.

## Figures and Tables

**Table 1:** Standard electrode potentials for the CO2RR into different products in aqueous solutions.

CO_2_ Reduction Reactions	Standard Electrode Potentials vs. NHE (V)
${CO}_{2}+e^{-}\to {CO}_{2}^{-}$	−1.90
${CO}_{2}+{2H}^{+}+{2e}^{-}\to HCOOH$	−0.61
${CO}_{2}+{2H}^{+}+{2e}^{-}\to CO+H_{2}O$	−0.53
${CO}_{2}+{4H}^{+}+{4e}^{-}\to HCHO+H_{2}O$	−0.48
${CO}_{2}+{6H}^{+}+{6e}^{-}\to CH_{3}OH+H_{2}O$	−0.38
${CO}_{2}+{8H}^{+}+{8e}^{-}\to CH_{4}+2H_{2}O$	−0.242

## Data Availability

Not applicable.

## Supplementary Materials

The following supporting information can be downloaded at: https://www.mdpi.com/article/10.3390/nano13111753/s1, Figure S1: (a) Cross-sectional view of SEM image of the nano-finger arrays from our previous work [27]. (b) Light intensity distribution of the nano-gap fingers from the electromagnetics simulation when using squared-shaped plasmonic nanostructures; Figure S2: Imaginary part of the epsilon of gold [31]; Figure S3: Electric field light intensity distribution of nano-fingers at (a) 638 nm and (b) 405 nm, and the light intensity distribution in logarithm scale of nano-fingers at (c) 638 nm and (d) 405 nm.
