# Supplementary material for: Plasmon-Enhanced Photocatalytic CO2 Reduction for Higher-Order Hydrocarbon Generation Using Plasmonic Nano-Finger Arrays
Source: Nanomaterials (Basel). 2023 May 27;13(11):1753. doi: 10.3390/nano13111753 (PMC10254476; doi:10.3390/nano13111753)
Supplement: Supplementary file 1 [file nanomaterials-13-01753-s001.zip › nanomaterials-2399046-supplementary.pdf]

Supporting Information

Plasmon-Enhanced Photocatalytic CO_2 Reduction for Higher-Order Hydrocarbon Generation Using Plasmonic Nano-Finger Arrays

Tse-Hsien Ou [1], Pan Hu [1], Zerui Liu [1], Yunxiang Wang [1], Sushmit Hossain [1], Deming Meng [1], Yudi Shi [1], Sonia Zhang [1], Boxin Zhang [2], Boxiang Song [3], Fanxin Liu [4], Stephen B. Cronin [1,5] and Wei Wu [1,*]

[1] Ming Hsieh Department of Electrical and Computer Engineering, University of Southern California, Los Angeles, CA 90089, USA
[2] Mork Family Department of Chemical Engineering and Material Science, University of Southern California, Los Angeles, CA 90089, USA
[3] Wuhan National Laboratory for Optoelectronics, Huazhong University of Science and Technology, Wuhan 430074, China
[4] Department of Applied Physics, Zhejiang University of Technology, Hangzhou 310023, China
[5] Department of Chemistry, University of Southern California, Los Angeles, CA 90089, USA
* Correspondence: wu.w@usc.edu

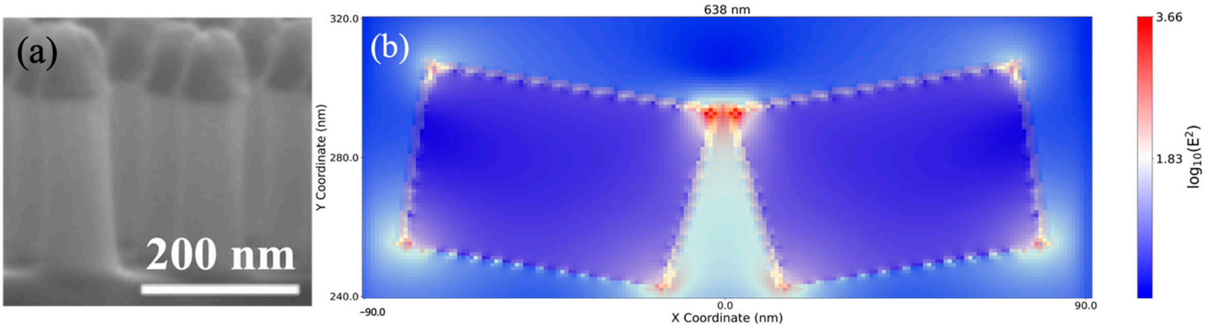

Figure S1. (a) Cross-sectional view of SEM image of the nano-finger arrays from our previous work [1]. (b) Light intensity distribution of the nano-gap fingers from the electromagnetics simulation when using squared-shaped plasmonic nanostructures.

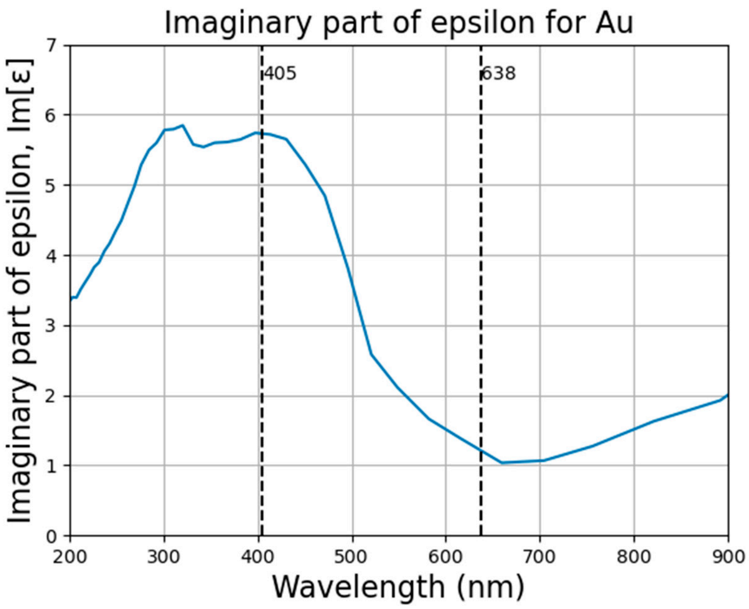

Figure S2. Imaginary part of the epsilon of gold [2].

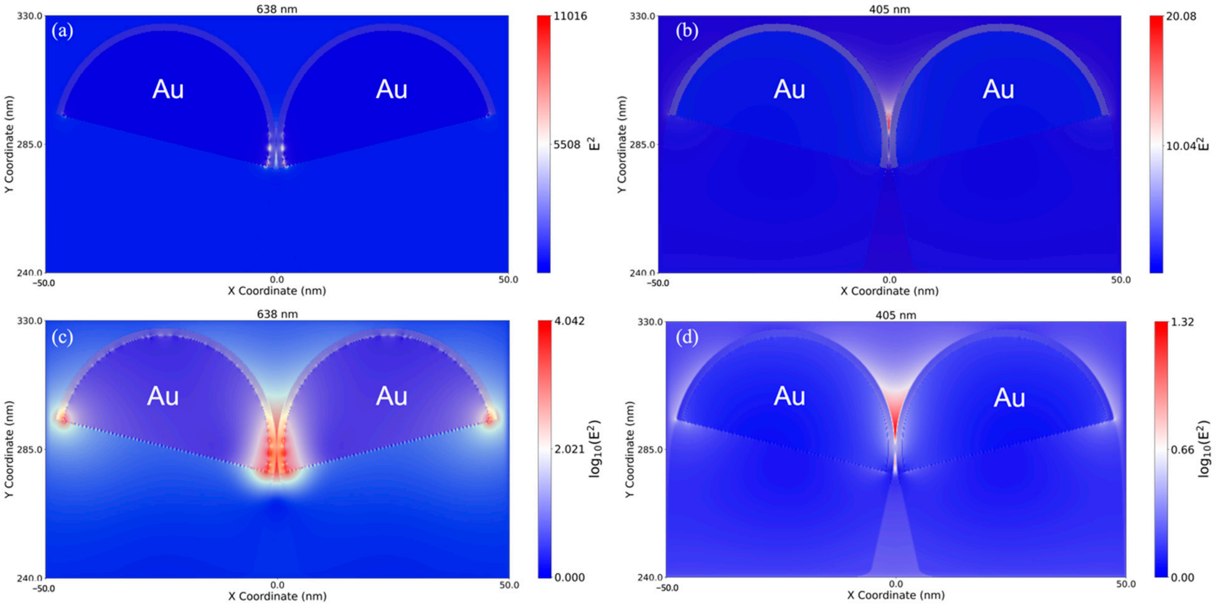

Figure S3. Electric field light intensity distribution of nano-fingers at (a) 638 nm and (b) 405 nm, and the light intensity distribution in logarithm scale of nano-fingers at (c) 638 nm and (d) 405 nm.

References
1. Song, B.; Yao, Y.; Groenewald, R.E.; Wang, Y.; Liu, H.; Wang, Y.; Li, Y.; Liu, F.; Cronin, S.B.; Schwartzberg, A.M.; et al. Probing Gap Plasmons Down to Subnanometer Scales Using Collapsible Nanofingers. *ACS Nano* **2017**, *11*, 5836–5843, doi:10.1021/acsnano.7b01468.
2. P. B. Johnson and R. W. Christy Optical Constant of the Nobel Metals. *Phys Rev B* **1972**, *6*, 4370–4379.
